# Levosimendan in Critical Illness: A Literature Review

**DOI:** 10.14740/jocmr1702w

**Published:** 2014-02-06

**Authors:** Charalampos Pierrakos, Dimitrios Velissaris, Federico Franchi, Luigi Muzzi, Menelaos Karanikolas, Sabino Scolletta

**Affiliations:** a Department of Intensive Care, Universite Catholique de Louvain, Mont-Godinne University Hospital, Yvoir 5530, Belgium; b Department of Internal Medicine, University of Patras School of Medicine, Patras, Greece; c Department of Medical Biotechnologies, University of Siena, Siena, Italy; d Department of Anesthesiology, Washington University School of Medicine, Campus Box 8054, 660 S. Euclid Avenue, St. Louis, MO, USA

**Keywords:** Levosimendan, Heart failure, Intensive care, Critical care, Renal failure, Liver failure, Heart failure, Sepsis, Myocardial infarction, Shock, Cardiogenic shock, Septic shock, Diastolic dysfunction, Coronary artery surgery, Valve surgery

## Abstract

Levosimendan, the active enantiomer of simendan, is a calcium sensitizer developed for treatment of decompensated heart failure, exerts its effects independently of the beta adrenergic receptor and seems beneficial in cases of severe, intractable heart failure. Levosimendan is usually administered as 24-h infusion, with or without a loading dose, but dosing needs adjustment in patients with severe liver or renal dysfunction. Despite several promising reports, the role of levosimendan in critical illness has not been thoroughly evaluated. Available evidence suggests that levosimendan is a safe treatment option in critically ill patients and may reduce mortality from cardiac failure. However, data from well-designed randomized controlled trials in critically ill patients are needed to validate or refute these preliminary conclusions. This literature review is an attempt to synthesize available evidence on the role and possible benefits of levosimendan in critically ill patients with severe heart failure.

## Introduction

Circulatory shock and peripheral hypo-perfusion are life-threatening manifestations of critical illness and are associated with high mortality [1]. Fluid administration serves as first-line therapy, but is often insufficient to improve patient’s condition, whereas beta-adrenergic agents increase cardiac output (CO) and organ perfusion through inotropic and chronotropic effects. Dobutamine, a beta-adrenergic agent commonly used in severe heart failure, increases CO, but also increases myocardial oxygen consumption, thereby increasing the risk of myocardial ischemia and ventricular dysfunction. Consequently, there are significant concerns regarding the balance of benefit vs. harm with dobutamine use in critically ill patients, and a recent systematic review suggests that dobutamine may increase mortality [2].

Levosimendan, the active enantiomer of simendan, is a calcium sensitizer that was developed for treatment of decompensated heart failure (DHF). Unlike other inotropic agents, levosimendan enhances myocardial contractility without increasing myocardial oxygen consumption, and its primary actions are independent of interactions with adrenergic receptors. Compared to beta-adrenergic agents, presumed advantages of levosimendan include its combined inotropic and vasodilation (inodilator) effect, efficacy on patients receiving beta-blockers and minimal effects on heart rate. Although levosimendan has been endorsed by the European Society of Cardiology for patients with acutely decompensated severe chronic heart failure [3], and a recent meta-analysis showed that levosimendan reduces mortality in critically ill patients [4], levosimendan is not widely used in intensive care units (ICUs). The aim of this review is to summarize and evaluate current evidence on the role of levosimendan in critically ill patients.

## Levosimendan: Mechanism of Action

Levosimendan is a calcium sensitizer and exerts its inotropic effect principally via binding to the Ca^++^ saturated troponin C of myocardial thin filament. This action results in stabilization of the Ca-bound conformation of troponin, thereby prolonging the actin-myosin interaction without altering cross-bridge cycling [5]. Although levosimendan inhibits phosphodiesterase III, its inotropic effect seems to depend almost entirely on its calcium sensitizing properties [6]. Consequently, in contrast to other inotropic agents, levosimendan does not increase calcium flux into the cell, and this could explain why levosimendan does not worsen myocardial diastolic dysfunction, and may actually improve diastolic function [7]. Interestingly, despite improved cardiac performance, levosimendan does not increase myocardial oxygen consumption and may increase myocardial oxygen supply through coronary vasodilation [8, 9].

Levosimendan causes vasodilation through its effect on K^+^ channels: it opens the K^+^ channels causing smooth muscle membrane hyperpolarization, thereby inhibiting calcium channels and promoting vasodilation [10]. However, because the vasodilator effect of levosimendan is observed at plasma concentrations higher than those needed for positive inotropic effects [11], its clinical significance is unclear. Levosimendan also induces vasodilation in other organs, including the myocardium, gastric mucosa [12], lungs [13], small intestine, liver and renal medulla [14]. As a result, organ perfusion is improved despite a small reduction of mean arterial pressure [9]. However, the clinical significance of levosimendan-related vasodilation has not been evaluated in critically ill patients, because studies evaluating levosimendan safety usually exclude patients with severe hypotension (systolic blood pressure (SBP) < 90 mmHg) (Table 1). The clinical consequences of levosimendan-related vasodilation should be evaluated taking in consideration concurrent improvement in cardiac performance.

**Table 1:** Studies Evaluating the Safety of Levosimendan in Critically Ill Patients

Study	Dose bolus	Dose infusion	Duration	Major adverse effects
Aidonidis et al. Cardiol Res Pract. 2011 [35]	0	0.05 - 0.2 µg/kg/min	72 h	No discontinuations due to adverse effects, two patients died of advanced heart failure.
Follath et al. Lancet. 2002 [38]	24 µg/kg	0.1 µg/kg/min	24 h	Hypotension, headache, hypokalemia
Kivikko et al. J Clin Pharmacol. 2002 [37]		0.05 - 0.1 µg/kg/min	7 days	No major adverse effects, no premature discontinuations
Moiseyev et al. Eur Heart J. 2002 [32]	6, 12, 24 µg/kg	0.1 - 0.4 µg/kg/min	6 h	Hypotension, myocardial rupture, headache, sinus tachycardia
Poelzl et al. Herz. 2008 [75]	6 - 12 µg/kg	0.07 - 0.2 µg/kg/min	24 h	Not reported
Silva-Cardoso et al. Rev Port Cardiol. 2009 [33]	12 µg/kg	0.05 - 0.2 µg/kg/min	24 h	Hypotension, hypokalemia

In addition to its inotropic and vasodilator effects, levosimendan has several other important effects, including anti-inflammatory effect [15, 16] and anti-apoptotic effects [15]. Levosimendan decreases pro-inflammatory cytokine production by diminishing transforming growth factor (TGF)-beta3 and Smad1, Smad2 and Smad3 expression [17]. In addition, simendans downregulate NF-kappaB-dependent transcription and decrease inducible NO synthase (iNOS) promoter activity, iNOS expression and nitric oxide (NO) production [18].

## Pharmacokinetics

Studies in healthy volunteers show that levosimendan pharmacokinetics are linear to the dose: the area under the plasma concentration-time curve (AUC) increases linearly and correlates with dose [19]. Levosimendan is 97-98% bound to plasma protein (mainly albumin) [20], has a small volume of distribution and its elimination from plasma is fast, with half-life being about 1 h [20]. Levosimendan is almost entirely metabolized by the liver before being eliminated by conjugation with glutathione, forming N-acetylated cysteine or cysteine-glycine. N-acetylated cysteine is excreted via the biliary route to the intestine, and is then eliminated by the kidneys [21].

A small amount of levosimendan is eliminated unchanged through diffusion in the intestine, where it is transformed by intestinal bacteria to OR-1855 [22]. Then, OR-1855 is absorbed and further acetylated by N-acetyltransferase (NAT2) to OR-1986, which has effects similar to the parent substance [23] and half-life 60 - 80 h [24]. Nearly 50% of OR-1986 is eliminated unchanged into urine, and the remainder is eliminated via other metabolic routes, including conversion back to OR-1855 [25].

Based on pharmacokinetic properties, levosimendan kinetics are influenced by: 1) albumin levels, 2) gastrointestinal function and intestinal bacterial flora, 3) liver function, 4) NAT2 activity and 5) renal function.

### Levosimendan and albumin levels

Hypoalbuminemia [26] and gastrointestinal dysfunction [27] are common in ICU patients, but their effects on levosimendan pharmacokinetics have not been studied. In a case report, low dose (0.07 µg/kg/min) levosimendan was effective in a patient with hypoalbuminemia (albumin level 3.1 mg/dL) [28]. In addition, two studies evaluating levosimendan on patients with low albumin levels due to chronic renal or liver failure showed that the mean unbound fraction of levosimendan did not differ compared to healthy people [29, 30]. However, albumin levels in these two studies were higher (3.8 g/dL ± 0.1 and 3.9 g/dL ± 0.1, respectively) compared to albumin levels commonly reported in critically ill ICU patients.

### Levosimendan and liver dysfunction

Liver dysfunction is common in ICU patients, and use of standard liver function tests may not be sufficient for evaluating liver function [31]. One small prospective study comparing levosimendan pharmacokinetics in 12 patients with Child-Pugh class B cirrhosis vs. 12 healthy subjects showed that OR-1896 elimination time was longer in cirrhosis patients [29]; however, this study did not include critically ill patients or patients with heart failure. Unfortunately, patients with liver dysfunction were excluded from two multi-center studies evaluating levosimendan safety [32, 33]; therefore data on levosimendan safety in patients with liver disease are very limited, and more work is needed in this area. Currently available data on the safety of levosimendan in critically ill patients are summarized in Table 1.

### Levosimendan and renal dysfunction

Acute renal failure is common in critically ill patients, and may affect the pharmacokinetics of levosimendan and its metabolites (OR-1896), due to reduced excretion. Levosimendan is not eliminated by hemodialysis, but levosimendan metabolites are dialyzable, yet clearance through dialysis is very slow (approximately 100 mL/min), and therefore short hemodialysis sessions may not have important effects [30]. An open label study on 12 patients with severe chronic renal failure and 13 patients with end-stage renal disease (ESRD) undergoing hemodialysis showed that, compared to healthy individuals, plasma half-life of levosimendan metabolites is prolonged by 50%, while the AUC and peak plasma concentrations are approximately twice as high in patients with severe renal dysfunction and those requiring hemodialysis [30]. However, we could not find studies evaluating levosimendan pharmacokinetics in patients with severe renal dysfunction in the setting of critical illness.

In conclusion, our literature review shows that levosimendan pharmacokinetics have not been well evaluated in critically ill patients, therefore more studies are needed to clarify this important issue.

## Levosimendan Dose

A wide range of levosimendan doses has been reported in critically ill patients (Table 1), with doses differing significantly between studies (bolus 0 - 24 µg/kg, continuous infusion 0.05 - 0.2 µg/kg/min) [4]. The hemodynamic effects of levosimendan seem to be dose-dependent [23], but are also influenced by severity of heart failure. A multi-center (35 centers) open label observational study in Brazil included 182 patients with DHF and left ventricular ejection fraction (LVEF) < 35% and the primary end point was hospital discharge without need for inotropic support after levosimendan infusion. One hundred and thirty-nine of 182 patients (76.4%) met the primary end point and were classified as responders, and hospital mortality was 14.8% (27 patients). In this study, 25 of 30 patients receiving beta-blockers responded well to levosimendan. In contrast, in the subset of 71 patients who had received beta-agonist inotropes for 48 h and had failed to improve, only 39 of 71 patients (56%) responded, thereby suggesting that patients with more severe heart dysfunction respond less favorably to levosimendan [34].

Several studies have evaluated levosimendan safety in patients with acute heart failure or decompensated chronic heart failure. Two studies evaluated levosimendan safety, with dose titrated based on tolerance and/or clinical effects [33, 35]. The RUSSLAN study, a randomized controlled trial (RCT) on 504 patients with left ventricular failure after acute myocardial infarction (MI) evaluated different levosimendan doses (bolus 6, 12 and 24 μg/kg, continuous infusion 0.1 - 0.2 μg/kg/h for 6 h) vs. placebo and showed higher frequency of hypotension and/or ischemia in the highest dose group, and significantly lower mortality in all levosimendan groups compared to placebo [32]. Interestingly, these studies did not report adverse effects after the bolus doses.

Most published studies evaluate levosimendan administration for 24 h and show rapid initial hemodynamic improvement that persists after levosimendan discontinuation [36]. In addition, one prospective study on 70 patients with decompensated chronic heart failure evaluated prolonged (up to 72 h) levosimendan infusion without a loading dose and showed significant reduction of brain natriuretic peptide (BNP) levels at 48 h and 72 h without major complications [35]. Similarly, a prospective non-randomized study on 24 patients with New York Heart Association (NYHA) III-IV heart failure evaluated a 7-day levosimendan infusion at 0.05 μg/kg/min (12 patients) or 0.1 μg/kg/min (12 patients) and showed that prolonged levosimendan infusion was well tolerated without premature discontinuations, but adverse effects included prolonged increase in heart rate and minor decrease in blood pressure [37]. However, the benefits, if any, of prolonged (> 24 h) levosimendan administration vs. conventional 24 h infusions have not been evaluated.

## Levosimendan vs. Other Inotropes

### Levosimendan vs. dobutamine

Levosimendan was compared to dobutamine in a multi-center RCT (LIDO study) on 213 patients with low CO heart failure (acute or decompensated chronic heart failure) requiring intravenous inotropes [38]. In this study, levosimendan was more effective than dobutamine in producing hemodynamic improvement: more patients in the levosimendan group (28% vs. 15%; P = 0.02) had CO increase by at least 30% and pulmonary-capillary wedge pressure decrease by 25% compared to baseline at 24 h. It is possible that levosimendan is more effective than dobutamine because it improves left ventricular diastolic function parallel with improvement of systolic function [39]. In general, levosimendan seems superior to dobutamine with regards to improving regional and systemic perfusion, possibly because of its greater vasodilator effect. An RCT on 40 patients with septic shock compared a 24 h infusion of levosimendan 0.2 µg/kg/min vs. dobutamine 5 µg/kg/min: dobutamine was comparable to levosimendan with regards to reduction of mean pulmonary artery pressure, right atrial pressure and pulmonary artery occlusion pressure, and increase of stroke index and cardiac index (CI), but regional and systemic perfusion were significantly higher in the levosimendan group [40].

Unfortunately, it is not clear whether the observed hemodynamic advantages of levosimendan over dobutamine result in improved survival. A secondary analysis of data from the LIDO study showed that patients treated with levosimendan had higher 180-day survival. In contrast, the SURVIVE study, a large (1,327 patients) multi-center (75 centers) international (9 countries) RCT showed that all-cause mortality at 6 months was 26% in the levosimendan group vs. 28% in the dobutamine group, and the difference was not significant [41]. In contrast, secondary analysis of the SURVIVE study data showed lower mortality with levosimendan in patients with decompensated chronic heart failure and in patients previously treated with beta-blockers [42]. However, levosimendan does not seem superior to dobutamine with regards to survival in patients with acute heart failure without history of chronic heart failure [43].

Levosimendan seems to have synergistic hemodynamic effects with dobutamine. A prospective study evaluated the effects of combining levosimendan with dobutamine on 18 patients hospitalized for NYHA class IV heart failure refractory to continuous 24-h infusion of dobutamine 10 µg/kg/min and furosemide 10 mg/h. Addition of levosimendan (6 µg/kg bolus followed by infusion at 0.2 µg/kg/min for 24 h) was well tolerated, increased CI by > 40% in 14 of 18 patients at the end of the combined infusion, and the observed improvement of CI persisted 1 week later. This study concluded that the levosimendan/dobutamine combination was more effective than dobutamine alone, without additional adverse effects in refractory heart failure [44]. These results suggest that levosimendan is probably not meant to replace dobutamine, but is a useful adjunct therapy in patients with heart failure refractory to dobutamine.

### Levosimendan vs. phosphodiesterase inhibitors

Experimental data suggest that levosimendan and phosphodiesterase inhibitors have similar efficacy in improving systolic cardiac function [45] and may exert similar cardioprotective effects [46]. However, levosimendan also improves diastolic cardiac function, whereas milrinone does not [46, 47].

An RCT on 30 patients with LVEF < 30% scheduled for elective cardiac surgery compared milrinone 0.5 µg/kg/min vs. levosimendan 0.1 µg/kg/min starting after the release of the aortic cross-clamp, while all patients also received dobutamine 5 µg/kg/min. In this study, patients in the levosimendan group required lower doses of norepinephrine (P = 0.007) had faster weaning from dobutamine, shorter duration of mechanical ventilation (P = 0.008) and no differences in adverse effects (hypotension, arrhythmia), ICU length of stay (LOS) or hospital LOS. With regards to mortality, there were three deaths in the milrinone vs. 0 deaths in the levosimendan group at 30 days, but this difference did not reach statistical significance [48].

Another RCT compared levosimendan (bolus 12 µg/kg over 10 min followed by infusion at 0.1 - 0.2 µg/kg/min for 24 h) vs. milrinone (bolus 50 µg/kg over 10 min followed by infusion of 0.3 - 0.5 µg/kg/min for 24 h) on 30 patients with type II diabetes and low LVEF who developed low cardiac output syndrome (LCOS) after cardiopulmonary bypass (CPB) for elective coronary artery bypass graft (CABG) surgery. This study showed significantly higher CI (P = 0.01) and mixed venous oxygen saturation (P < 0.001) and significantly lower oxygen extraction ratio (P = 0.03), pulmonary capillary wedge pressure (PCWP) (P = 0.04) and systemic vascular resistance (SVR) (P = 0.01) in the levosimendan group compared to the milrinone group [49].

Levosimendan has also been compared to enoximone in an RCT on 32 patients with persistent cardiogenic shock (shock despite successful reperfusion therapy, administration of inotropes and fluids and support with intra-aortic balloon pump (IABP)) after acute MI. Levosimendan was given as 12 µg/kg over 10 min loading dose followed by 0.1 µg/kg/min infusion for 50 min and 0.2 µg/kg/min infusion over 23 h, whereas enoximone was given as 0.5 µg/kg over 30 min bolus followed by 2 - 10 µg/kg/min infusion titrated to hemodynamic response for a median of 5.1 days. In this study, although hemodynamic changes did not differ significantly between the two groups, survival was higher in the levosimendan group (69% with levosimendan vs. 37% with enoximone, P = 0.023) [50].

## Levosimendan in Critically Ill Patients

### Levosimendan in cardiac surgery

Morbidity after cardiac surgery has increased in recent years and, despite improved cardioplegic protection, postoperative LCOS remains a problem, largely because cardiac surgery is increasingly utilized in older, higher risk patients. LCOS consists of transient ventricular dysfunction related to global myocardial ischemia and reperfusion injury, is also known as myocardial stunning and is associated with high morbidity and mortality. In one meta-analysis, cardiac surgery patients who received levosimendan perioperatively had lower mortality compared to patients treated with placebo, dobutamine or milrinone (4.7% vs. 12.7%, P = 0.003), whereas another meta-analysis included 139 cardiac surgery patients from five RCTs and showed that levosimendan has cardioprotective effects, as evidenced by lower postoperative troponin levels in patients receiving levosimendan. However, these finding are based on relatively small number of patients, and need to be confirmed in larger studies [51, 52].

One RCT evaluating levosimendan (10 µg/kg loading dose followed by 0.1 µg/kg/min for 24 h) vs. dobutamine (starting at 5 µg/kg/min) as monotherapy in 137 patients with LCOS after elective CABG showed fewer complications and significantly lower mortality (9% vs. 25%, P < 0.05) in the levosimendan group [53].

Levosimendan for primary graft failure after heart transplantation has been reported in two publications originating from the same center in Germany [54, 55]. Levosimendan was given as 24-h infusion at 0.1 µg/kg/min in 12 patients with primary graft failure, resulted in rapid improvement of heart function and 30 day survival was 92% [55].

Prophylactic levosimendan has also been evaluated in cardiac surgery: an RCT evaluated levosimendan use (bolus 12 µg/kg followed by infusion at 0.2 µg/kg/min) immediately after induction of general anesthesia vs. placebo in 60 patients with LVEF < 50% undergoing CABG surgery, and showed that a primary weaning from CPB was successful in 22 patients (73%) in the levosimendan group vs. 10 patients (33%, P = 0.002) in the placebo group [56]. Another prospective study in patients with LVEF < 35% undergoing cardiac surgery under CPB compared prophylactic levosimendan (bolus 12 µg/kg followed by infusion at 0.1 µg/kg/min for 24 h) starting after induction of anesthesia vs. IABP starting 16 - 18 h before surgery, and showed higher CI and lower troponin levels 6 h after surgery and significantly shorter ICU LOS in the levosimendan group [57].

Similarly, another RCT on 252 patients with LVEF < 25% undergoing CABG under CPB, compared preoperative levosimendan (loading dose 10 µg/kg followed by infusion at 0.1 µg/kg/min for 23 h) vs. placebo and showed significantly lower incidence of LCOS (7.1% vs. 20.8%, P < 0.05), lower requirement for IABP (6.3% vs. 30.4%, P < 0.05) and lower mortality (3.9% vs. 12.8%, P < 0.05) in the levosimendan group [58]. Another small retrospective study compared 10 high-risk cardiac surgery patients (Euroscore > 6) with severe left ventricle (LV) dysfunction who received prophylactic levosimendan (bolus 24 µg/kg followed by infusion at 0.1 µg/kg/min for 24 h) with 12 historical controls and showed that patients who received levosimendan had higher CI, shorter hospital LOS and lower 30-day mortality compared to patients who received dobutamine and milrinone [59].

Furthermore, an RCT on 106 patients undergoing elective CABG surgery evaluated levosimendan (24 µg/kg as slow IV bolus 10 min before CPB) vs. placebo and showed lower troponin values (P = 0.0001), reduced need for inotropic support and significantly shorter duration of mechanical ventilation and ICU LOS (P = 0.01) in the levosimendan group [60].

However, a retrospective matched-control study in patients with LVEF < 30% undergoing cardiac surgery concluded that prophylactic levosimendan infusion starting before weaning from CPB resulted in significantly lower mean arterial pressure and need for higher doses of norepinephrine, without any benefit in CI, mixed venous oxygen saturation, or 30-day mortality [61].

Although the inotropic and vasodilator effects of levosimendan are a concern in patients with severe valve disease, particularly aortic stenosis [62], a recent report described successful preoperative levosimendan use in two adults with severe aortic stenosis [63], and a few studies have evaluated the use of levosimendan in heart valve surgery. A small RCT from Finland on 24 patients undergoing aortic valve replacement with or without CABG showed that patients who received levosimendan at 0.2 µg/kg/min for 24 h starting after induction of anesthesia required more norepinephrine during surgery and less nitroprusside after surgery, but maintained their pre-CPB LVEF and avoided myocardial stunning [64]. Similarly, a large RCT from a different center in Finland evaluated levosimendan (24 µg/kg bolus over 30 min) starting immediately after induction of anesthesia and continuing at 0.2 µg/kg/min for 24 h vs. placebo in 200 patients with normal preoperative cardiac function undergoing elective isolated valve or combined valve-CABG surgery [65]. This study showed reduced heart failure but increased need for vasopressors and no difference in 6-month mortality in the levosimendan group.

In conclusion, the optimal strategy for levosimendan administration (prophylactic vs. use in LCOS) in cardiac surgery is matter of discussion [66]. Early levosimendan administration may be more effective [67], but the issue has not been resolved and deserves further evaluation.

### Levosimendan in cardiogenic shock

Because levosimendan causes vasodilation, cardiogenic shock has been exclusion criterion in studies evaluating levosimendan safety, and therefore levosimendan has not been adequately studied in cardiogenic shock. In the BELIEF study, hypotension (SBP < 90 mmHg) before levosimendan administration was a good predictor for absence of response to levosimendan treatment [34]. However, published data suggest that levosimendan is well tolerated in patients with cardiogenic shock after acute MI [68, 69] and its hemodynamic effects are comparable to those of IABP [70], but the timing of levosimendan administration remains matter of controversy. One prospective observational study compared early (in the cardiac catheterization suite at the time of IABP initiation) vs. late (persistent cardiogenic shock despite successful reperfusion therapy, IABP and optimized fluid administration) levosimendan administration and showed no benefit in short term (< 30 days) or long term (> 30 days) mortality with early levosimendan administration [71]. Late levosimendan administration significantly increased CI without hemodynamic deterioration in patients with persistent cardiogenic shock (no improvement of CI after reperfusion treatment, IABP, optimized fluid or dobutamine administration) [72, 73], but hemodynamic improvement did not improve survival [72]. To this day, there are no RCTs evaluating levosimendan in cardiogenic shock secondary to acute MI.

Experimental data in dogs show that levosimendan improves right ventricular function in load-induced acute right ventricular failure [74]. Three studies evaluating levosimendan in cardiogenic shock due to bi-ventricular acute heart failure showed improved right ventricular function [75-77], presumably due to decreased pulmonary vascular resistance and improved right ventricular systolic function. Furthermore, the observed improvement of right ventricular systolic function (as measured with right ventricular cardiac power index (CPI)) was related to survival [77]. To this date, there are no studies evaluating levosimendan in cardiogenic shock secondary to isolated right heart failure.

Levosimendan could potentially be effective in other types of cardiogenic shock, such as in fulminant myocarditis [78], in patients with takotsubo cardiomyopathy [79, 80] and in cardiomyopathy after cardiac arrest, but there are no prospective clinical trials evaluating the role of levosimendan in these settings.

### Levosimendan in sepsis

Sepsis and septic shock have complex pathophysiology, including cardiovascular dysfunction characterized by signs of distributive shock and of septic cardiomyopathy consisting of bi-ventricular myocardial contractility impairment and diastolic dysfunction. Available evidence suggests that cardiac failure in septic shock is not always corrected by vasoactive and inotropic agents or fluid therapy [81].

Experimental studies show that levosimendan improves systolic and diastolic myocardial function in sepsis, whereas clinical studies have shown that levosimendan improves cardiac function in patients with septic shock. Consequently, levosimendan could be a valuable adjunct (or replacement) in the treatment of sepsis-related myocardial dysfunction with catecholamines, but there are no guidelines for levosimendan use in sepsis. A pilot RCT on 35 patients with septic shock and acute respiratory distress syndrome (ARDS), but without signs of right heart failure showed that continuous infusion of levosimendan (0.2 µg/kg/min for 24 h) decreased pulmonary resistance and improved right ventricular function (decreased telosystolic volume, increased EF) compared to placebo [82]. Yet, it is unclear whether the observed improvement of right ventricular function and reduction of pulmonary vascular resistance is due to direct levosimendan effect on the right ventricle or to improved LV function.

Levosimendan could also be effective in patients with sepsis-related myocardial dysfunction who do not respond to dobutamine: an RCT on 28 patients with septic shock and left ventricular dysfunction showed hemodynamic improvement with levosimendan (0.2 µg/kg/min) even in patients who had not responded to dobutamine [83]. These results suggest that levosimendan could be an alternative option in cases where dobutamine is not effective, but this possible use needs further evaluation.

### Levosimendan in renal dysfunction and renal failure

The long-term impact of levosimendan on renal function has not been well defined. An RCT on 40 patients with advanced chronic heart failure listed for heart transplantation evaluated the effect of levosimendan on renal function, and showed that patients receiving levosimendan had lower serum creatinine and higher creatinine clearance 3 months after levosimendan administration [84]. Other reports also suggest that levosimendan can have beneficial effects with regards to renal function. A RCT by Bragadottir [85] compared postoperative infusion of levosimendan (loading 12 µg/kg followed by infusion at 0.1 µg/kg/min) vs. placebo in the cardiothoracic ICU on 30 patients who had cardiac surgery. In this study, levosimendan increased renal blood flow by 12% (P < 0.05) and glomerular filtration rate by 21% (P < 0.05). Similarly, an RCT by Hou et al compared a 24-h infusion of levosimendan vs. placebo in 66 patients with LVEF < 40% and DHF, and showed that levosimendan transiently improved renal dysfunction, and this improvement persisted for at least 14 days [86]. A study by Yilmaz showed that, compared to dobutamine, levosimendan resulted in improved renal function in heart failure patients requiring inotropic therapy [76], and a case report of a 14-year-old patient suggested that levosimendan is useful in cases of resistant acute heart failure with arterial hypertension and ESRD [87]. However, it is important to remember that levosimendan doses should be reduced when used for congestive heart failure in patients with severe renal insufficiency [30]. A recent consensus statement by 25 scientists from 15 European countries acknowledged that most reports on levosimendan use show improvement of renal function in heart failure, sepsis and cardiac surgery, but also voiced caution with interpretation of these findings due to variability in study design, and also because the largest heart failure study (REVIVE I and II) did not show improved renal function [88]. Clearly, although available data suggest that levosimendan can be useful in patients with renal dysfunction and heart failure, the role of levosimendan in this patient population needs further study.

## Conclusion

Levosimendan is a positive inotropic agent with many potential applications in critically ill patients. However, indications, contraindications and optimal use of levosimendan in the ICU have not been adequately evaluated. Although one meta-analysis and several clinical studies report positive results with use of levosimendan in critically ill patients, we believe that currently available evidence cannot support safe conclusions regarding the role of levosimendan in the ICU, and data from large, well-designed RCTs are needed to validate these preliminary results.

## Abbreviations

ARDS: acute respiratory distress syndrome
AUC: area under the concentration-time curve
CABG: coronary artery bypass grafting
CI: cardiac index
CO: cardiac output
CPB: cardiopulmonary bypass
DHF: decompensated heart failure
ESRD: end-stage renal disease
IABP: intra-aortic balloon pump
ICU: intensive care unit
iNOS: inducible NO synthase
LCOS: low cardiac output syndrome
LOS: length of stay
LV: left ventricle
LVEF: left ventricular ejection fraction
MI: myocardial infarction
NAT: N-acetyltransferase
NO: nitric oxide
NYHA: New York Heart Association
PCWP: pulmonary capillary wedge pressure
RCT: randomized controlled trial
SBP: systolic blood pressure
SVR: systemic vascular resistance
TGF: transforming growth factor
